# Characteristics and Clinical Analysis of Dysphagia Caused by Pontine Infarction: A Video Fluoroscopic Swallowing Study

**DOI:** 10.1002/brb3.70957

**Published:** 2025-10-20

**Authors:** Yuemin Gao, Pengna Hao, Nuan Yang, Zhengmao Xiang, Zhengfan Li, Linxi Li, Xiaona Cheng, Dan Tao, Xiaoguang Lang, Zhijuan Liang, Fang Hu, Xuehai Lv

**Affiliations:** ^1^ Department of Rehabilitation Medicine Handan Central Hospital Handan China; ^2^ Graduate School Hebei Medical University Shijiazhuang China; ^3^ Graduate School Chengde Medical University Chengde China; ^4^ Graduate School Hebei North University Zhangjiakou China; ^5^ Academy of Geography, Sociology, and International Studies Hong Kong Baptist University Hong Kong China

**Keywords:** dysphagia, swallowing angiography

## Abstract

**Objective:**

To investigate the characteristics of a video fluoroscopic swallowing study (VFSS) in patients with dysphagia caused by different parts of pontine infarction and to analyze the relationship between the severity of dysphagia and the parts of pontine infarction.

**Methods:**

This study included 60 patients diagnosed with acute pontine infarction in the Department of Neurology and Rehabilitation Medicine of Handan Central Hospital from August 2022 to August 2023. Pontine infarction was divided into an upper pontine infarction group, middle pontine infarction group, and lower pontine infarction group according to the distribution characteristics of diffusion‐weighted imaging (DWI). The VFSS evaluation results of patients with dysphagia brought on by infarction in different areas of the pons were calculated and analyzed to summarize the characteristics of dysphagia. The odified barium swallow impairment profile (MBSImP) was used to assess the severity of swallowing impairment in patients with pontine infarction at different locations.

**Results:**

Compared with the upper and middle pontine infarction groups, the lower pontine infarction group was more prone to oropharyngeal swallowing disorder, and the incidence of lip closure, tongue control during bolus hold, oral residue, initiation of the pharyngeal swallow, laryngeal elevation, and tongue base retraction showed statistically significant differences among the three patient groups (*p* < 0.05). Compared with the suprapontine and middle infarct groups, the lower pontine infarct group had a higher MBSImP score in the oral and pharyngeal stages of swallowing disorders and a heavier degree of lip closure, tongue control during bolus hold, oral residue, initiation of the pharyngeal swallow, laryngeal elevation, and tongue base retraction, with statistical significance (*p* < 0.05).

**Conclusion:**

Patients with subpontine infarction with dysphagia had relatively independent biological characteristics.

## Graphical abstract

This study investigates dysphagia characteristics in pontine infarction patients using video fluoroscopic swallowing study (VFSS), categorizing infarcts into upper, middle, and lower pons regions. Key findings reveal that lower pontine infarction patients exhibit significantly more severe oropharyngeal dysfunction, with higher incidences of impaired lip closure, tongue control, laryngeal elevation, and tongue base retraction compared to upper/middle infarcts (*p* < 0.05). VFSS‐based modified barium swallow impairment profile (MBSImP) scores confirmed worse oral/pharyngeal stage impairments in lower pons lesions, highlighting their distinct clinical profile. The results underscore the critical role of infarct location in dysphagia severity, advocating for targeted rehabilitation strategies.

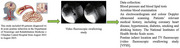

## 1 Introduction

Ischemic cerebral infarction in China is known for its high morbidity and mortality rates (Wu et al. 2015). Pontine infarction, a common type of infarction in the posterior circulation, accounts for 7.3% of all cerebral infarctions (Oh et al. 2012). Swallowing dysfunction is a significant complication following cerebral infarction, with an incidence ranging from 51% to 73% in China. Common swallowing abnormalities observed in clinical practice include salivation, choking when drinking water, and lingual muscle weakness (Xiao et al. 2020, Zhang et al. 2019, Bai et al. 2020).

The physiological processes involved in swallowing are intricate (Ji et al. 2013). Neurophysiological evidence suggests the presence of a swallowing ‘central pattern generator’ (CPG) located in the medulla oblongata (Xu and Wu 2020, Huang et al. 2016). Unlike the medulla oblongata and supratentorial structures, the pons has no control over the pharyngeal center, often leading to dysphagia (Suntrup et al. 2015).

Currently, X‐ray swallowing function examinations are widely used as the ‘gold standard’ for evaluating and diagnosing swallowing disorders. However, these methods have limitations, such as exposure to radiation and the inability to provide real‐time dynamic assessment of the swallowing process (Chinese Society of Neurology, Cerebrovascular Group, Chinese Society of Neurology 2018). Common treatments for dysphagia include dietary modifications, swallowing therapy, and, in severe cases, the use of feeding tubes (Bradley 1991). While these treatments can be effective, they often do not address the underlying neurological deficits that contribute to dysphagia.

This study aims to explore the characteristics of swallowing angiography in patients with pontine infarction and analyze the relationship between the severity of dysphagia and the location of pontine infarction. Specifically, we seek to understand how different parts of the pons, each with distinct functional roles, contribute to the manifestation of dysphagia following pontine infarction. This knowledge could help inform more targeted and effective treatment strategies for patients with pontine infarction‐related dysphagia.

## 2 Methods

### 2.1 Study Participants

This research was conducted as a prospective study aimed at exploring the differences in the manifestations of dysphagia following pontine infarction in different parts of the pons. Inpatients diagnosed with acute pontine infarction in the Department of Neurology and Rehabilitation Medicine of Handan Central Hospital from August 2022 to August 2023 were included. This study was reviewed and approved by the hospital ethics committee, and the patients voluntarily participated and provided informed consent.

Inclusion criteria: (a) The patient met the diagnostic criteria specified in the *Chinese Guidelines for the Diagnosis and Treatment of Acute Ischemic Stroke 2018* (Chinese Society of Neurology, Cerebrovascular Group, Chinese Society of Neurology 2018) and was confirmed as having acute pontine infarction by brain magnetic resonance imaging (MRI) examination. (b) The patient had dysphagia after improved drinking water screening. (c) Patients with abnormalities in the improved drinking water test agreed to undergo video fluoroscopic swallowing study (VFSS) for consistency for swallowing. (d) First onset or duration of disease was < 7 days (Chinese Society of Neurology, Cerebrovascular Group, Chinese Society of Neurology 2018), with stable vital signs, and could actively cooperate with early treatment and evaluation. (e) The patient provided signed informed consent.

Exclusion criteria: (a) Television fluoroscopy for swallowing could not be performed. (b) Patients with critical illness or cognitive and communication disabilities who could not cooperate with the assessment. (c) Patients with other diseases that had affected their swallowing function in the past.

### 2.2 Sample Size Calculation

To ensure the robustness and reliability of our study findings, we conducted a sample size calculation prior to conducting the study. The calculation was based on the primary outcome measure, the modified barium swallow impairment profile (MBSImP) score, which was used to detect a clinically significant difference in swallowing impairment between different pontine infarction groups.

We aimed to detect a medium effect size (Cohen's *d* = 0.5) with a power of 80% (1−*β* = 0.8) and a significance level of *α* = 0.05, based on the formula for sample size calculation in a two‐group comparison (assuming equal variances and equal sample sizes in each group). Accordingly, we needed at least 32 patients per group to achieve the desired power and significance level. Considering potential dropouts and to ensure sufficient statistical power, we decided to include 20 patients in each of the three pontine infarction groups for a total of 60 patients.

### 2.3 Diagnostic Standards for Dysphagia

Dysphagia was diagnosed using an improved water swallowing screening test, which is a simple and widely used method to identify patients at risk of swallowing disorders. The test was conducted as follows: (1) The patient was asked to sit upright and take a sip of 1 mL of water. (2) If the patient showed no signs of choking or difficulty swallowing, they were then asked to drink 3 mL of water. (3) If still no abnormalities were observed, the patient was asked to drink 5 mL of water. (4) Finally, the patient was asked to drink 30 mL of water in one go, and the time taken and any signs of difficulty were closely observed.

### 2.4 Data Collection

All of the enrolled patients underwent blood pressure and blood lipid tests, a routine blood examination, an electrocardiogram, and color Doppler ultrasound scanning. Patients’ relevant medical history, including coronary heart disease, hypertension, diabetes, and smoking and drinking history. The National Institutes of Health Stroke Scale score, pontine infarct location, and TV fluoroscopy (video fluoroscopic swallowing study [VFSS]) results were also recorded.

### 2.5 Research Methods

**FIGURE 1:**
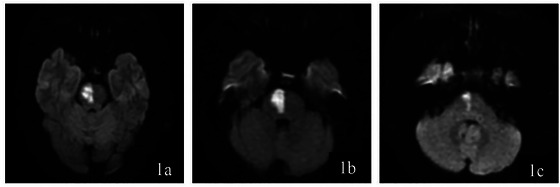
a–c: Diffusion‐Weighted Imaging (DWI) scans illustrating distinct pontine infarction locations. **(a)** Displays an upper pontine infarction characterized by the hyperintense signal in the upper section of the pons, **(b)** Depicts a middle pontine infarction with the infarcted area appearing as a hyperintense region in the middle section of the pons, and **(c)** Shows a lower pontine infarction, indicated by the bright signal in the lower part of the pons.

Magnetic resonance imaging examination: The cerebral infarction location was reported by the imaging physician according to the location of the responsible lesion, and the diagnosis was acute pontine infarction. Infarct sites are classified according to imaging results: According to an imaging study of the pons conducted by Bradley et al. (Bradley 1991), the pons is divided into three parts: the upper, middle, and lower sections. (Figure 1)

Improved water swallowing test screening (Brodsky et al. 2016): The test was conducted within 24 h of admission. The patient took a sitting position and drank 1 mL of water first. If no issues were experienced, they then drink 3 and 5 mL of water, respectively. If no abnormality was observed, the patient was asked to drink 30 mL of water at one time and was closely observed to record drinking time and drinking status, and the results were combined with clinical judgment as to whether the patient had a swallowing disorder.

Scoring system for the improved water swallowing test: Score 1: The patient could drink 30 mL of water in one go without choking or coughing. Score 2: The patient could drink 30 mL of water in one go but coughed during or after swallowing. Score 3: The patient had to drink 30 mL of water in more than one go but did not cough. Score 4: The patient had to drink 30 mL of water in more than one go and coughed during or after swallowing. Score 5: The patient was unable to swallow 30 mL of water. Patients who scored 2 or higher on this test were considered to have dysphagia and were included in the study.

**FIGURE 2:**
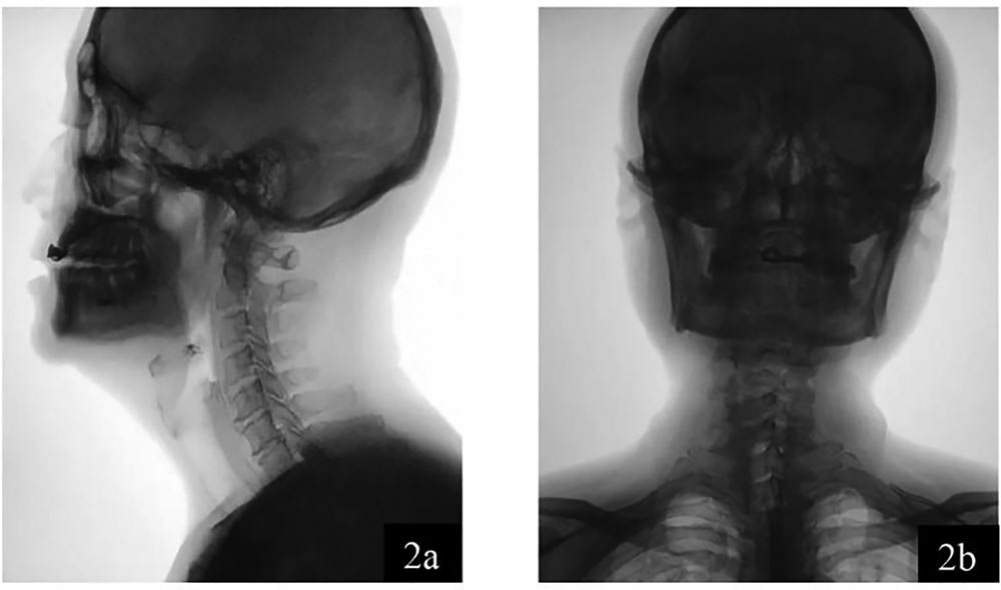
VFSS images depicting swallowing function from two perspectives. **(a)** Lateral view of a patient during the VFSS, illustrating the side profile of the oral cavity, pharynx, and upper esophageal sphincter as a bolus is swallowed. This view is essential for observing the lateral movements of the tongue, the position of the larynx, and the coordination of the pharyngeal walls and **(b)** Anteroposterior view of the same patient, providing a frontal perspective of the oral cavity, pharynx, and upper esophagus. This view allows for the assessment of the symmetry of the swallowing mechanism and the opening of the pharyngoesophageal segment during swallowing.

Video fluoroscopic swallowing study (Tomita et al. 2018): This was conducted within 48 h of admission. The test was prepared and performed as follows: (a) A 60% w/v barium sulfate suspension was prepared by mixing 200 mg of barium sulfate with 286 mL of water. Then, 166 mL of this suspension was combined with 5 g of Ourdiet Swallow (Ourdiet Swallow Solid Beverage Guangzhou Urdiet Biotechnology Co.) to create a 3% thickened liquid. Then, 30 mL of the barium sulfate suspension was mixed with 60 mL of the 3% thickened liquid to produce a 2% semi‐thickened food. Then, 30 mL of this barium sulfate suspension was mixed with another 30 mL of the medium‐thickened food to form a low‐thickened food at a concentration of 1%. For a solid presentation, use a cookie as a base for the visible portion containing the concentrated, thickened liquid. (b) Patient preparation: The patients were seated in upright and lateral positions, respectively, for examination and video recording. (c) Test sequence: The tests were performed in the order of medium consistency, low consistency, high consistency, and solid state. A radiologist and speech therapist jointly analyzed the video results and recorded the characteristics of swallowing abnormalities after reaching an agreed conclusion. (Figure 2) (d) Evaluation criteria: The VFSS abnormal results were mainly recorded as follows: oral impairment domain (lip closure, tongue control during bolus hold, bolus preparation/mastication, bolus transport/lingual motion, oral residue, initiation of the pharyngeal swallow); pharyngeal impairment domain (soft palate elevation, laryngeal elevation, anterior hyoid excursion, epiglottic movement, laryngeal vestibular closure, pharyngeal stripping wave, pharyngeal contraction, pharyngoesophageal segment opening, tongue base retraction, pharyngeal residue); esophageal impairment domain: esophageal clearance (upright position). Based on the imaging findings, these three‐impairment domains were described as an oral, pharyngeal, or esophageal stage, and the total scores of the oral and pharyngeal stages were calculated.

### 2.6 Statistical Methods

The SPSS 25.0 software was used for data analysis. The normality of the measurement data was assessed using the Shapiro–Wilk test. Data that followed a normal distribution were represented by mean ± standard deviation (x ± s), and between‐group comparisons were performed using parametric tests (one‐way ANOVA for comparisons among three groups). Data that did not conform to a normal distribution were represented by median and quartile ranges (M [P25, P75]), and nonparametric tests were adopted. Specifically, the Kruskal–Wallis *H* test was used for comparisons among the three groups, and the Mann–Whitney *U* test was used for post‐hoc pairwise comparisons. For categorical variable data, the chi‐square test was used when the expected frequency (T) was not below 5 in more than 20% of the cells; otherwise, Fisher's exact test was applied. In this study, *p* < 0.05 was used to indicate statistical significance. For multiple comparisons among groups, the Bonferroni correction was applied to control the family‐wise error rate, resulting in a corrected significance level of *α* = 0.017.

## 3 Results

Baseline characteristics of patients: A total of 60 patients with acute pontine infarction were included in this study, including 20 patients each in the upper, middle, and lower pontine infarction groups. There was no statistical significance in the comparison of general data among the three groups (*p* > 0.05). (Table 1)

**TABLE 1:** Patient's baseline condition.

Components	Upper pontine infarction group N_1_ = 20	Middle pontine infarction group N_2_ = 20	Lower pontine infarction group N_3_ = 20	Inter‐group comparison	
	(%:n/N_1_)	(%:n/N_2_)	(%:n/N_3_)	X^2^/F	*p*
Age (years)	65.10 ± 9.94	68.20 ± 8.66	61.85 ± 9.16	2.348	0.105b
Male	15 (75)	14 (70)	13 (65)	0.476	0.788a
Diabetes	9 (45)	12 (60)	10 (50)	0.934	0.627a
Hypertension	13 (65)	13 (65)	18 (90)	4.261	0.119a
Coronary heart disease	4 (20)	6 (30)	5 (25)	0.533	0.766a
Dyslipidemia	18 (90)	17 (85)	17 (85)	0.424	> 0.999a
Smoking history	3 (15)	6 (30)	4 (20)	1.346	0.630a
Drinking history	2 (10)	4 (20)	4 (20)	1.035	0.749a
mRS	1.20 ± 0.48	1.30 ± 0.51	1.45 ± 0.57	0.532	1.123
Revascularization treatment (%)	5 (25)	7 (35)	6 (30)	1.143	0.798
NIHSS	5.15 ± 2.08	5.30 ± 2.43	4.75 ± 2.17	0.324	0.725b

*Note*: a means Chi‐square test, *p* > 0.05. b means analysis of variance, *p* > 0.05.

**FIGURE 3:**
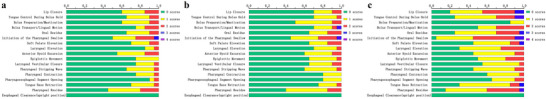
**(a)** The incidence of various symptoms in patients with upper pontine infarction and dysphagia, **(b)** The incidence of various symptoms in patients with mid pontine infarction and dysphagia, and **(c)** The incidence rate of various symptoms in patients with lower pontine infarction and swallowing disorders.

Video fluoroscopic swallowing study evaluation results in the three groups: The rates of lip closure, tongue control during bolus holds, oral residue, initiation of the pharyngeal swallow, laryngeal elevation, and tongue base retraction in the three patient groups showed statistically significant differences overall (*p* < 0.05). Post‐hoc pairwise comparisons revealed that the rates of lip closure, tongue control during bolus hold, oral residue, initiation of the pharyngeal swallow, laryngeal elevation, and tongue base retraction were higher in the lower pontine infarction group compared to the upper pontine infarction group (*p* < 0.017). Similarly, these rates were also higher in the lower pontine infarction group compared to the middle pontine infarction group (*p* < 0.017) (Table 2, Figure 3).

**TABLE 2:** Comparison of VFSS evaluation results in different regions of pontine infarction groups.

Components	Upper pontine infarction group N_1_ = 20	Middle pontine infarction group N_2_ = 20	Lower pontine infarction group N_3_ = 20	Inter‐group comparison				
	(%:n/N_1_)	(%:n/N_2_)	(%:n/N_3_)	X^2^	*P*	*P_ab_*	*P_bc_*	*P_ac_*
Lip closure	8 (40)	6 (30)	16 (80)*#	11.200	0.004	0.507	0.001	0.010
Tongue control during bolus hold	7 (35)	6 (30)	15 (75)*#	9.777	0.008	0.736	0.004	0.011
Bolus preparation/mastication	8 (40)	10 (50)	3 (15)	5.714	0.057	0.525	0.018	0.077
Bolus transport/lingual motion	5 (25)	6 (30)	10 (50)	3.077	0.215	0.723	0.197	0.102
Oral residue	7 (35)	7 (35)	15 (75)*#	8.543	0.014	>0.999	0.011	0.011
Initiation of the pharyngeal swallow	10 (50)	11 (55)	19 (95)*#	10.950	0.004	0.752	0.003	0.001
Soft palate elevation	6 (30)	7 (35)	11 (55)	2.917	0.233	0.736	0.204	0.110
Laryngeal elevation	3 (15)	6 (30)	14 (70)*#	13.678	0.001	0.451	0.011	< 0.001
Anterior hyoid excursion	9 (45)	8 (40)	11 (55)	0.938	0.626	0.749	0.342	0.527
Epiglottic movement	5 (25)	7 (35)	10 (50)	2.727	0.256	0.490	0.337	0.102
Laryngeal vestibular closure	5 (25)	7 (35)	9 (45)	1.758	0.415	0.490	0.519	0.185
Pharyngeal stripping wave	7 (35)	8 (40)	11(55)	1.765	0.414	> 0.999	0.342	0.204
Pharyngeal contraction	5 (25)	4 (20)	8 (40)	2.134	0.344	> 0.999	0.168	0.311
Pharyngoesophageal segment opening	2 (10)	4 (20)	7 (35)	3.543	0.190	0.661	0.288	0.127
Tongue base retraction	6 (30)	5 (25)	14 (70)*#	10.011	0.007	0.723	0.004	0.011
Pharyngeal residue	11 (55)	12 (60)	1 7(85)	4.650	0.098	0.749	0.077	0.038
Esophageal clearance (upright position)	0 (0)	0 (0)	0 (0)	0.000	> 0.999	—	—	—

**FIGURE 4:**
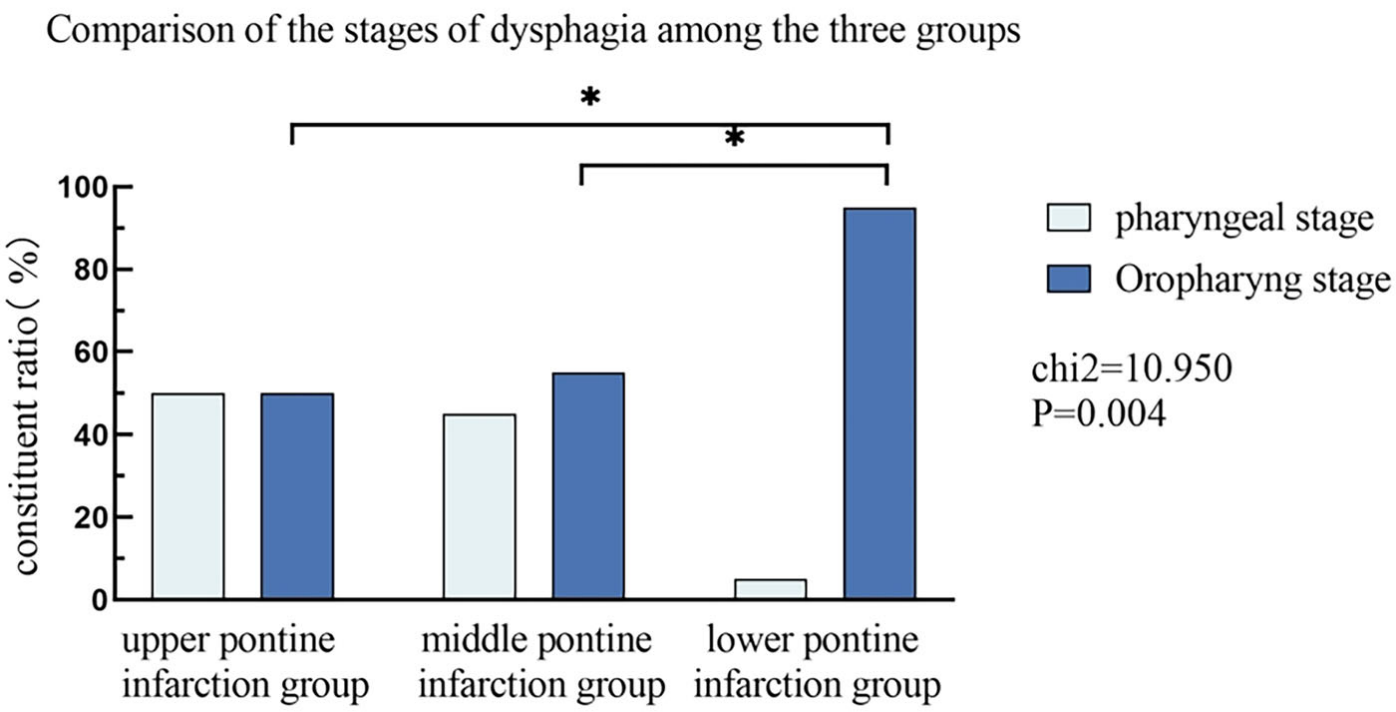
Comparison of three stages of swallowing disorders.

Comparison of the stages of dysphagia among the three groups: the oropharyngeal stage accounted for 50.00% in the upper pontine infarction group, 55.00% in the middle pontine infarction group, and 95.00% in the lower infarction group, and there were statistical differences in the proportions among the three groups (*p* < 0.05). Following pairwise comparison, a statistically significant difference was observed in the proportion of oropharyngeal dysphagia between the upper and lower pontine infarction groups (*p* = 0.001, *p* < 0.017), as well as between the lower and middle pontine infarction groups (*p* = 0.003, *p* < 0.017) (Table 3, Figure 4).

**TABLE 3:** Comparison of stages for swallowing disorders.

Groups	Total(cases)	Pharyngeal stage [Cases (%)]	Oropharyng stage [Cases (%)]	Chi‐square test				
				X^2^	*P*	*P_ab_*	*P_bc_*	*P_ac_*
Upper pontine infarction group	20	10 (50)	10 (50)	10.950	0.004	0.753	0.003	0.001
Middle pontine infarction group	20	9 (45)	11 (55)					
Lower pontine infarction group	20	1 (5)	19 (95)*#					

*Note: P_ab_* represents the comparison between the upper group and middle group of pontine infarction; *P_bc_* is a comparison between the group with middle pontine infarction and the group with lower pontine infarction; *P_ac_* is a comparison between the upper and lower pontine infarction groups. *Compared with the upper pontine infarction group, the difference was statistically significant (*P_ac_* < 0.017). # Compared with the middle pontine infarction group, there was a statistical difference (*P_bc_* < 0.017).

**FIGURE 5:**
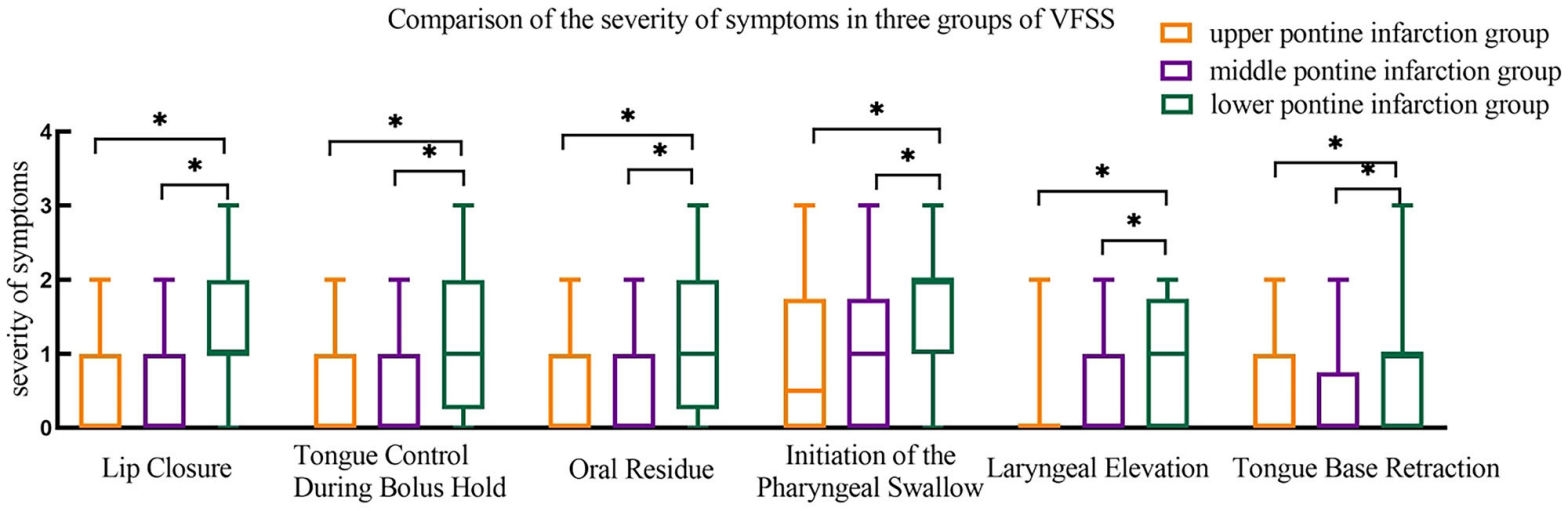
Swallowing disorder symptom score in different regions of the pontine infarction group.

Comparison of the severity of VFSS symptoms among the three groups: Lip closure, tongue control during bolus holds, oral residue, initiation of the pharyngeal swallow, laryngeal elevation, and tongue base retraction in the lower pontine infarction group were higher than in the upper and middle pontine infarction groups, and the differences among the three groups were statistically significant (*p* < 0.05) (Figure 5).

**FIGURE 6:**
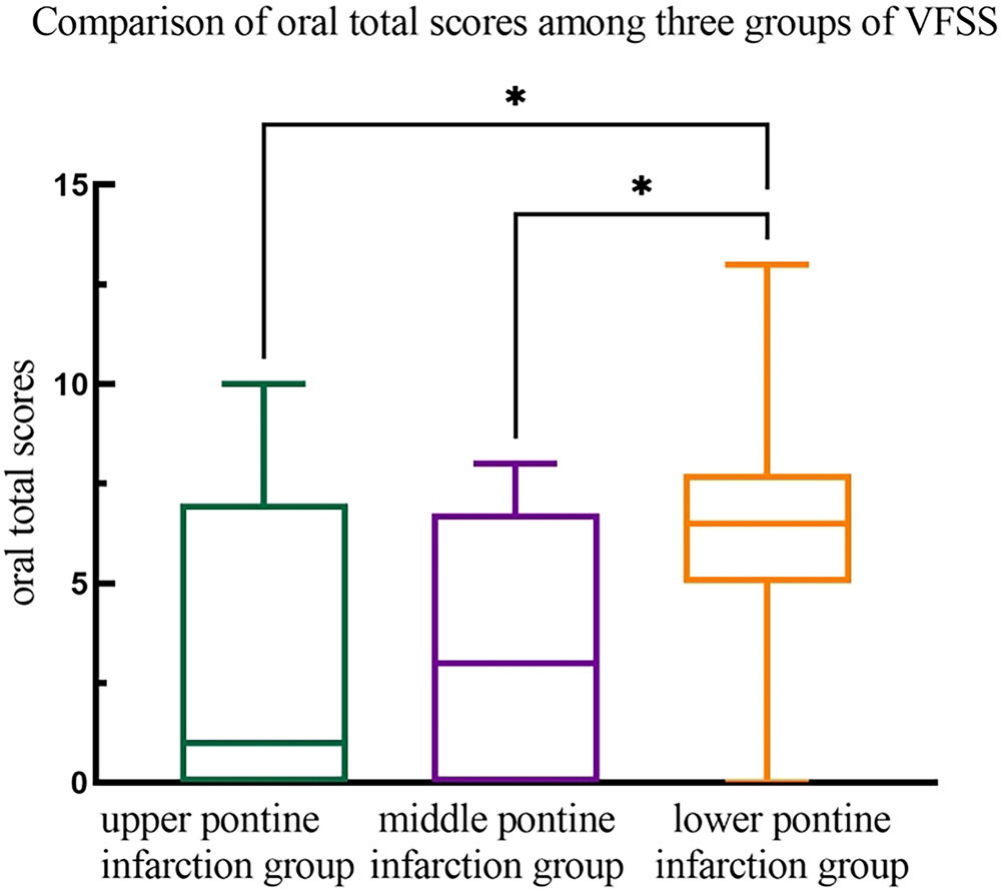
Total score of swallowing disorders in the oral cavity of patients with different locations of pontine infarction.

Comparison of the severity of swallowing disorder among the three groups: The oral total scores for the upper, middle, and lower pontine infarction groups were 1.00 (0.00, 7.00), 3.00 (0.00, 6.75), and 6.50 (5.00, 7.75), respectively. Significant differences were observed among these groups (F = 8.773, *p* = 0.012). The lower pontine infarction group had significantly higher scores than the upper (*p* = 0.032) and middle (*p* = 0.030) pontine groups (Table 4, Figure 6).

**TABLE 4:** Comparison of total oral scores among three groups of VFSS.

Groups	M (P25, P75)	Chi‐square test	
		*H*	*P*
Upper pontine infarction group	1.00 (0.00, 7.00)		
Middle pontine infarction group	3.00 (0.00, 6.75)	8.773	0.012
Lower pontine infarction group	6.50 (5.00, 7.75)*#		

*Note*: *Compared with the upper pontine infarction group, the difference was statistically significant. # Compared with the middle pontine infarction group, there was a statistical difference.

**FIGURE 7:**
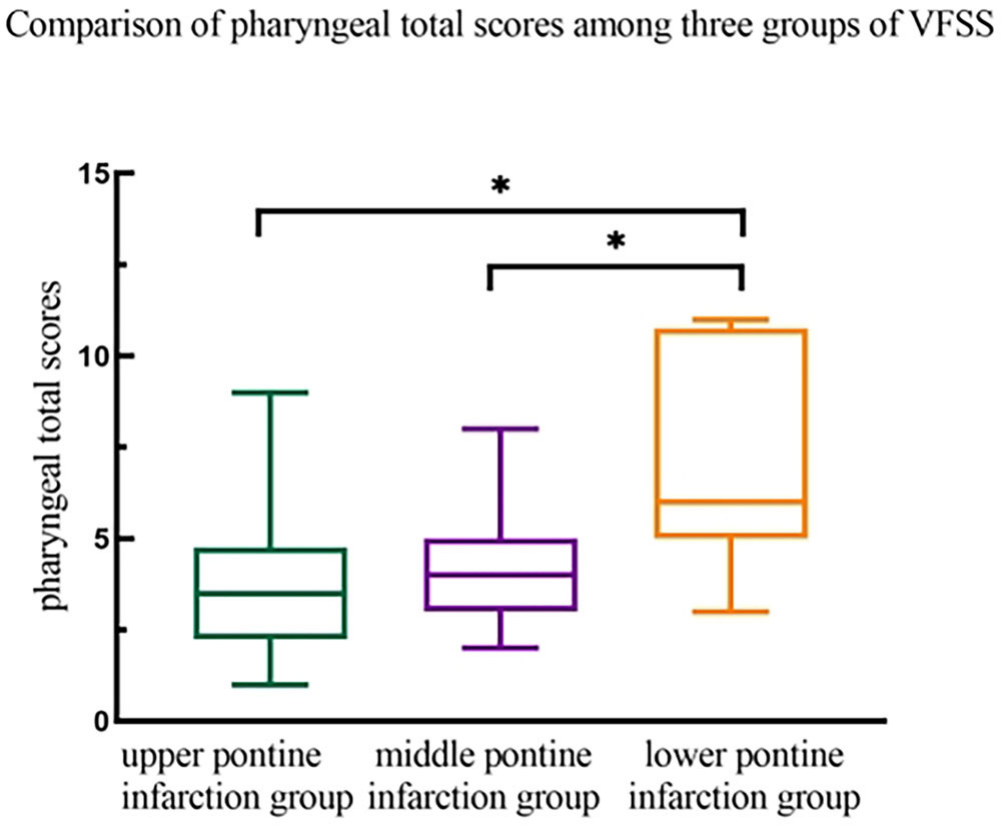
Total score of swallowing disorders in the pharyngeal region of different regions with pontine infarction.

The pharyngeal total scores were 3.50 (2.25, 4.75) for the upper pontine group, 4.00 (3.00, 5.00) for the middle pontine group, and 6.00 (5.00, 10.75) for the lower pontine group. Significant differences were found among these groups (F = 21.671, *p* < 0.001). The lower pontine infarction group showed significantly higher scores compared to the upper (*p* < 0.001) and middle (*p* = 0.001) pontine groups (Table 5, Figure 7).

**TABLE 5:** Comparison of total pharyngeal scores among three groups of VFSS.

Groups	M(P25, P75)	Chi‐square test	
		*H*	*P*
Upper pontine infarction group	3.50 (2.25, 4.75)		
Middle pontine infarction group	4.00 (3.00, 5.00)	21.671	< 0.001
Lower pontine infarction group	6.00 (5.00, 10.75)*#		

*Note*: * Compared with the upper pontine infarction group, the difference was statistically significant. # Compared with the middle pontine infarction group, there was a statistical difference.

## 4 Discussion

This study investigated the characteristics of dysphagia in patients with different pontine region infarctions using VFSS. The key findings revealed that the lower pontine infarction group was more prone to oropharyngeal swallowing disorders, with significantly higher incidences of lip closure, tongue control during bolus hold, oral residue, initiation of the pharyngeal swallow, laryngeal elevation, and tongue base retraction. These were better in the lower pontine infarct group compared with the upper and middle pontine infarction groups (*p* < 0.05). Furthermore, the lower pontine infarction group exhibited higher MBSImP scores in both the oral and pharyngeal stages of swallowing disorder, indicating a more severe degree of dysphagia (*p* < 0.05). These results suggest that the location of pontine infarction significantly influences the manifestation and severity of dysphagia, with the lower pontine region being particularly associated with more severe swallowing impairments. This study provides valuable insights into the clinical management of patients with pontine infarction and highlights the need for targeted assessments and interventions based on the specific location of the infarct.

Although the pons is not the primary swallowing center, studies have found the incidence of dysphagia in patients with isolated pons infarction to be 34.2% (Lapa et al. 2017) and 23.7%–72.1% in the acute stage (Maeshima et al. 2012), which is considered to be related to the pivotal role of the pons.

The patients included in this study who had dysphagia caused by pontine infarction were found to have impaired swallowing function in the pharyngeal stage. Conversely, for patients with dysphagia with lower pontine infarction, this mainly involved the oropharyngeal stage. In a study conducted by Wang et al. (2009), pontine infarction was found to have primarily resulted in swallowing abnormalities in the pharyngeal stage, while medullary infarction resulted in mixed oropharyngeal swallowing abnormalities, with statistical significance between the two classifications (*p* < 0.05). Neither category showed swallowing abnormalities in the oral stage only, which is similar to the current study's results. The oral phase is regulated by autonomous consciousness, while the pharyngeal and oesophageal phases are controlled by autonomic nerve reflexes (Tang 2020). Because the cortical bulbar tract is involved in the triggering of active swallowing, the structure of the cortical bulbar tract in the upper part of the pons is not tightly distributed. In contrast, the structure of the cortical bulbar tract in the lower part of the pons is tightly arranged. In addition, the hypoglossal nerve nucleus is located in the medulla oblongata, which is innervated by the bulbar tract of one side of the cortex, and injury to the hypoglossal nerve will affect the control of oral food (Yuan 2022). Relevant studies have also reported cases of adverse tongue movement caused by pontine infarction (Ku et al. 2012), which may also explain why hypoglossal infarction is more likely to lead to dysphagia in the oropharyngeal stage.

The VFSS results of patients with pontine infarction at different locations were analyzed in this study. The composition ratio of lip closure, tongue control during bolus hold, oral residue, initiation of the pharyngeal swallow, laryngeal elevation, and tongue base retraction in the lower pontine infarction group was higher than in the upper and middle pontine infarction groups. Vlašković et al. (2022) pointed out in their study that patients in the lower pontine infarction group generally experienced facial paresis or supranuclear lingual or palatal paralysis, which is consistent with our findings. The facial nerve nucleus is located in the lower part of the pons (Deng and Li 2014), while the oral dysphagia of patients with upper and middle pons infarction is mostly considered to be caused by central facial palsy (Zhang 2022), which is consistent with the results of a study conducted by Guo et al. (2016) indicating muscle damage on the affected side of patients with unilateral brainstem stroke as being relatively serious. Nakao et al. (2019) found that CPG impairment could affect the sequential order of the swallowing process. When the sensory nucleus of the trigeminal nerve is damaged after a pontine stroke, the sensory sensitivity of the tongue and pharynx to the food mass decreases (Vaiman et al. 2005), and the receptors in the mouth, tongue, and pharynx cannot reach the threshold value to trigger the swallowing reflex. The upper area of the NTS is located in the lower part of the pons, which can receive taste sensations from the facial nerve and glossopharyngeal nerve. Afferent signals from oropharyngeal mucosal nerve endings ascend to the nucleus of the solitary tract (NTS), while efferent signals from the swallowing cortex descend to the same region. NTS damage impairs sensory integration, causing pharyngeal‐phase swallowing delays, muscle incoordination, and weak/abnormal contractions (Wang 2017).

The severity of dysphagia in patients with pontine infarction at different locations was analyzed in this study. Based on the outcomes, compared with the suprapontine and midpontine infarction groups, patients in the lower pontine infarction group had higher total oral scores and stronger degrees of dysphagia. Among these, the degree of lip closure, tongue control during bolus hold, oral residue, and initiation of the pharyngeal swallow in the lower pontine infarction group was higher than in the upper and middle pontine infarction groups. In a study completed by Shu et al. (2023), the results showed that the severity of swallowing in patients with stroke was closely related to defects in the corticobulbar tract, while other studies confirmed that a lower pontine infarction indicated more obvious damage to the pyramidal tract (Oh et al. 2012, Li et al. 2020). The entire swallowing process requires thorough mixing with saliva. The superior salivary nucleus is located in the sub‐bridge of the brain (Wang 2022), and damage to this nucleus will affect the lubrication and dilution of food by saliva, resulting in swallowing difficulties. This also explains the greater degree of dysphagia during the oral stage in the lower infarction group.

The current study found that, compared with the upper and middle pontine infarction groups, the lower pontine infarction group had a higher pharyngeal total score and a heavier degree of swallowing disorder. The degree of laryngeal elevation and tongue base retraction in the lower pontine infarction group was higher than in the upper and middle pontine infarction groups. A study by Li et al. (2022) found that stroke patients with pharyngeal dysphagia exhibited delayed activation of the hyolaryngeal complex in the brainstem compared to normal swallowers and oral dysphagia patients. This suggests impaired neural reflex activity controlling hyolaryngeal movement during the pharyngeal phase of swallowing. During normal swallowing, the tongue rears back and pulls the hyoid bone, providing the basis for laryngeal elevation. Lower pontine infarction resulted in abnormal conduction of central and cerebral nerves innervating the hyoid and laryngeal fixation muscle groups, leading to the inability of the normal movement of the hyoid‐laryngeal complex to be regulated (Urabe et al. 2019). The lower part of the pontine is closer to the medulla oblongata, which has a more significant effect on tongue movement, and the upper part of the nucleus of the solitary tract can easily affect the function of CPG after injury. This can also lead to more severe swallowing disorders in cases of lower pontine infarction.

The sample size in the current study was relatively small, which may have limited the generalizability of the findings. Additionally, no detailed analysis was conducted of the specific swallowing condition of each patient to observe individual trait image markers. Future research should focus on expanding the sample size to further validate these findings and explore the underlying mechanisms in greater detail. This study is important for guiding future diet types for patients and will be the focus of future research. We anticipate expanding the sample data, enhancing the research content, and further developing this topic in the future.

Additionally, this study focused on investigating the characteristics of VFSS in patients with dysphagia caused by pontine infarction at different locations and analyzing the relationship between infarction sites and dysphagia severity. The decision not to include healthy controls was based on the following considerations: (1) The primary research objective was to compare swallowing function differences among pontine infarction subgroups (upper, middle, and lower), rather than making comparisons with healthy populations; (2) VFSS is primarily used to assess abnormal swallowing patterns in dysphagia patients, such as oral residue and delayed pharyngeal swallow initiation—pathological manifestations that do not exist in healthy individuals; (3) Performing VFSS on healthy individuals raises ethical concerns regarding radiation exposure and presents practical challenges in volunteer recruitment; (4) Assessment tools like the MBSImP are specifically designed to quantify dysphagia severity, with scoring criteria that are not applicable to healthy populations without swallowing disorders. Future studies may consider incorporating noninvasive techniques such as surface electromyography or ultrasound to supplement baseline data on normal swallowing function.

## Author Contributions

Gao YM and Hao PN conceived and design the study. Yang N, Xiang ZM, Li ZF and Li LX collected the data. Cheng XN, Tao D, Lang XG and Liang ZJ helped the data analysis and statistics. Hu F and Lv XH took part in drafting the manuscript. All authors read and approved the final manuscript.

## Ethics Statement

This study was conducted strictly following the ethical requirements of medical studies in the Helsinki Declaration (2013 Revision) and approved by the Ethics Committee of Handan Central Hospital.

## Consent

All participants signed a written informed consent before entering the study.

## Peer Review

The peer review history for this article is available at https://publons.com/publon/10.1002/brb3.70957

## Data Availability

All data generated or analyzed during this study are included in this published article.
